# A genome-wide association study and genomic prediction for *Phakopsora pachyrhizi* resistance in soybean

**DOI:** 10.3389/fpls.2023.1179357

**Published:** 2023-05-29

**Authors:** Haizheng Xiong, Yilin Chen, Yong-Bao Pan, Jinshe Wang, Weiguo Lu, Ainong Shi

**Affiliations:** ^1^ Department of Horticulture, University of Arkansas, Fayetteville, AR, United States; ^2^ Sugarcane Research Unit, Untied State Department of Agriculture – Agriculture Research Service (USDA-ARS), Houma, LA, United States; ^3^ Henan Academy of Crops Molecular Breeding, National Centre for Plant Breeding, Zhengzhou, China

**Keywords:** GWAS, soybean, disease resistance, genomic prediction, *Phakopsora pachyrhizi*

## Abstract

Soybean brown rust (SBR), caused by *Phakopsora pachyrhizi*, is a devastating fungal disease that threatens global soybean production. This study conducted a genome-wide association study (GWAS) with seven models on a panel of 3,082 soybean accessions to identify the markers associated with SBR resistance by 30,314 high quality single nucleotide polymorphism (SNPs). Then five genomic selection (GS) models, including Ridge regression best linear unbiased predictor (rrBLUP), Genomic best linear unbiased predictor (gBLUP), Bayesian least absolute shrinkage and selection operator (Bayesian LASSO), Random Forest (RF), and Support vector machines (SVM), were used to predict breeding values of SBR resistance using whole genome SNP sets and GWAS-based marker sets. Four SNPs, namely Gm18_57,223,391 (LOD = 2.69), Gm16_29,491,946 (LOD = 3.86), Gm06_45,035,185 (LOD = 4.74), and Gm18_51,994,200 (LOD = 3.60), were located near the reported P. pachyrhizi R genes, Rpp1, Rpp2, Rpp3, and Rpp4, respectively. Other significant SNPs, including Gm02_7,235,181 (LOD = 7.91), Gm02_7234594 (LOD = 7.61), Gm03_38,913,029 (LOD = 6.85), Gm04_46,003,059 (LOD = 6.03), Gm09_1,951,644 (LOD = 10.07), Gm10_39,142,024 (LOD = 7.12), Gm12_28,136,735 (LOD = 7.03), Gm13_16,350,701(LOD = 5.63), Gm14_6,185,611 (LOD = 5.51), and Gm19_44,734,953 (LOD = 6.02), were associated with abundant disease resistance genes, such as *Glyma.02G084100*, *Glyma.03G175300*, *Glyma.04g189500*, *Glyma.09G023800*, *Glyma.12G160400*, *Glyma.13G064500*, *Glyma.14g073300*, and *Glyma.19G190200*. The annotations of these genes included but not limited to: *LRR* class gene, *cytochrome 450*, cell wall structure, *RCC1, NAC, ABC* transporter, *F-box* domain, etc. The GWAS based markers showed more accuracies in genomic prediction than the whole genome SNPs, and Bayesian LASSO model was the ideal model in SBR resistance prediction with 44.5% ~ 60.4% accuracies. This study aids breeders in predicting selection accuracy of complex traits such as disease resistance and can shorten the soybean breeding cycle by the identified markers

## Introduction

Soybean brown rust (SBR) is one of the most devastating fungal diseases of soybean (*Glycine max*) (Hartman et al., 2005). It first emerged around 1900 as a threat to soybean production in China and Japan and has since spread globally, in part due to human activities and meteorological phenomena (Hartman et al., 1991). The disease arrived in Africa and the Pacific Islands in the 1980s and 1990s and later reached the American continents in the 2000s (Miles et al., 2004). The risk of SBR attracted more attention with the disease outbreak in China in 1975 and in Brazil in 2001, that caused 10 billion US dollar losses in each country (Yorinori et al., 2005; Godoy et al., 2016). Comparing to the native American rust pathogen (*Phakopsora meibomiae*), the exotic one (*Phakopsora pachyrhizi*) was much more aggressive and caused an epidemic on soybean in South America and spread to North America (Pivonia and Yang, 2004).

Soybean plants are susceptible to SBR at any stage of growth and development and *Phakopsora pachyrhizi* can quickly spread over a long-range through wind-borne urediniospores (Isard et al., 2005). Therefore, it is important to develop control strategies for controlling SBR. Currently, the SBR can be managed by applying fungicides and employing specific cultivation practices (Levy, 2005). However, considering the high cost and the harm to non-target beneficial fungi, a more economic, safer, and environmental friendly solution is to raise varieties’ own resistance by developing new resistance lines through breeding or engineering (Bromfield and Hartwig, 1980). In the past 30 years, the well-known *Rpp 1*–*7* genes were mapped to chromosome 3, 6, 16, 18, and 19 (Garcia et al., 2008; Pandey et al., 2011; Li et al., 2012; Kashiwa et al., 2020). However, *Rpp* genes were race-specific and provided resistance exclusively to specific *P. pachyrhizi* isolates. Currently, there is no resistant soybean genotype that can ward off all known *P. pachyrhizi* isolates (Childs et al., 2018a). In addition, *Rpp* gene-mediated resistance can be overcome swiftly in the field due to pathogen’s adaptation and evolution to resistant host (Godoy and Meyer, 2020). Pyramiding three or more *Rpp* genes into one genotype to obtain broader and/or more durable resistance has been reported on other crops like wheat or barley, but traditional breeding is still time-consuming and may introduce unwanted traits (Childs et al., 2018a). Another promising strategy for sustainable and effective SBR resistance is to utilize alternative R gene combinations and dynamic turnover in the field (Childs et al., 2018a). However, the identity of these *Rpp* genes needs to be revealed (Gebremedhn et al., 2020). Under the current conditions, it is also impractical to rely only on several major genes or combinations of these genes to control the SBR disease in field production.

In addition to major genes, many recent molecular studies have revealed more disease-resistant pathways in soybeans (Childs et al., 2018b). The resistance usually occurs in the form of signals, transcription factors, NB-LRR, or secondary metabolites (Gebremedhn et al., 2020; Waheed et al., 2021). They usually improve not only the resistance to a particular pathogen but the overall resistance of the plant as well. In addition, many minor resistance/tolerance genes are widely distributed throughout the whole soybean genome and exhibit partial defense response (PDR) to SBR (Langenbach et al., 2016). PDR is characterized by low infection frequency, long-lasting latency, small lesions, and reduced spore production per uredinium (Langenbach et al., 2016). At the molecular level, their specific functions are sometimes very similar or overlapping to the context components; however, they are more complex and obscure (Langenbach et al., 2016). Screening for or silencing susceptibility is another strategy that can provide durable disease resistance in breeding, because of susceptible (S) gene function either as susceptibility factors or suppressors of plant defense, thus potential targets of fungal effectors (De Wit, 1992). For example, absence of the S gene *Mlo* in barley results in an incompatibility interaction with *Blumeria graminis hordei* that resembles nuclear hormone receptors (Büschges et al., 1997; Lucas, 2020). However, the identification and mapping of S gene are more difficult than those of major R genes by linkage mapping, and only one [Cys(2)His(2) zinc finger TF palmate-like pentafoliata1, PALM1] would classify as a S gene so far (Uppalapati et al., 2012).

Molecular marker-assisted selection (MAS) has been applied in soybean breeding to accelerate the development of disease-resistant varieties, and the GWAS is of vital help to MAS (He et al., 2014). Comparing with linkage mapping, GWAS can not only find the major genes, but also has the incomparable ability to map and identify the minor and S genes. Moreover, since the mapping populations such as natural population and multi-parent advanced generation inter-cross, contain more diversity, the markers developed have more universal applicability (Visscher et al., 2012). So far, only one SBR-related GWAS has been reported by Chang et al. (2016), who used GWAS to discover five SBR-related loci from USDA germplasm. Genomic selection (GS) has gained popularity in recent years in modern and large-scale crop breeding programs. GS can predict the breeding value of an individual plant based on its genotype to estimate the field performance of the plant, whereas MAS relies on the detection of a few QTLs using a simple linear model. Therefore, molecular breeding would shift from marker-assisted selection to genomic selection, as the genetic architecture of resistance changes from a single major R gene to multiple minor diffusion gene architectures (Poland and Rutkoski, 2016). Additionally, GS has been reported to be a useful tool in soybean breeding to predict a wide range of traits, including both agronomic and quality traits (Lorenz et al., 2011). However, no research has been done with respect to investigating GS accuracy for SBR resistance/tolerance.

The objectives of this study were to identify SBR resistance-associated SNP markers and to characterize the ability of genomic prediction in order to use SNP markers in selecting soybean breeding lines highly resistant to SBR.

## Materials and methods

### Plant materials and phenotyping

SBR disease scores and phenotyping data of 3,082 soybean accessions (**Table S1**) were downloaded from the USDA GRIN website (https://npgsweb.ars-grin.gov/gringlobal/method?id=492634) (Miles et al., 2006). Based on the website, a greenhouse study was initiated. Soybean plants of 3,082 accessions were spray-inoculated between the first and second trifoliate stage with a mixture of urediniospores (60,000 spores per ml) from four *Phakopsora pachyrhizi* isolates, incubated overnight in a dew chamber at 22–25°C, and placed in a greenhouse at 20–25°C for 14 days. Disease severity was evaluated on the first trifoliate leaves for most accessions; however, the unifoliate leaves were evaluated for a few accessions due to slow germination (Miles et al., 2006). Based on the symptom and lesion development, a disease severity scale of 1 to 5 was used, where 1 = no visible symptom, 2 = light infection: only a few small (less than 1 cm) rust lesion present on the leaves, 3 = light to moderate infection: moderately sized (1–2 cm) rust lesion present on a limited number of leaves, 4 = moderate to severe infection: large (greater than 2 cm) rust lesion present on a significant number of leaves, and 5 = severe infection: nearly all leaves are covered in large rust lesion, and the disease is causing a significant damage to the plant growth (Walker et al., 2011).

### Genotyping

The Soy50K SNP Infinium Chips (Song et al., 2013) and a total of 42,292 SNPs across 3,082 soybean accessions were downloaded from the Soybase at https://www.soybase.org/snps/download.php. SNPs with >10% missing data, >8% heterozygous genotypes, and <10% minor allele frequencies (MAF) were removed, and 30,314 SNPs were included in the GWAS study.

### Population structure and genetic diversity

LEA is an R package for population structure and genomic signature analysis of local adaptation. The inference algorithms used by R are based on a fast version of structure available from the R package LEA (Frichot and François, 2015). The structure analysis identifies K clusters by measuring an optimum ΔK based on the SNP data provided. A preliminary analysis was performed in multiple runs by inputting successive values of K from 2 to 20. After an optimum K was determined, each soybean accession was assigned to a cluster (Q) based on the probability that the accession belonged to that cluster. The cut-off probability for the assignment to a cluster was 0.5. Based on the optimum K, a bar plot with “Sort by Q” was obtained to visualize the population structure among the 3,082 accessions. Phylogenetic relationships among the accessions was generated by TASSEL 5.2.13 and phylogenetic tree was drawn using R package: Phytologist and Phytools (Revell, 2012). During the drawing of the phylogeny trees, the population structure and the cluster information were imported for the combined analysis of genetic diversity. For sub-tree of each Q (cluster), the shape of “Node/Subtree Marker” and the “Branch Line” was drawn using the same color scheme of the STRUCTURE analysis.

### Linkage disequilibrium analysis and SNP based haplotype blocks

TASSEL 5.0 (Bradbury et al., 2007) was used to calculate the linkage disequilibrium (LD) for all pairwise loci. Only SNPs with a minor allele frequency (MAF) greater than 0.10 and less than 10% missing data were included in the LD estimation process. Haplotype blocks (HAP) were estimated by Plink 2.0 (Purcell et al., 2007) within 200kb (r^2^ ≈ 0.4), and a minimum threshold value 0.05 for MAF.

### Genome-wide association study

GWAS was performed using the Generalized Linear Model (GLM), Mixed Linear Model (MLM) (Jiang and Nguyen, 2021), Compressed Mixed Linear Model (CMLM), Multiple Loci Mixed Model (MLMM) (Wen et al., 2018), Settlement of MLM Under Progressively Exclusive Relationship (SUPER) (Wang et al., 2014), Fixed and Random Model Circulating Probability Unification (FarmCPU) (Liu et al., 2016), and Bayesian-information and Linkage-disequilibrium Iteratively Nested Keyway (BLINK) (Wang et al., 2014) in R software GAPIT 3 (Genomic Association and Prediction Integrated Tool version 3) (Wang and Zhang, 2021; Lipka et al., 2012; https://zzlab.net/GAPIT/index.html; https://github.com/jiabowang/GAPIT3) by setting PCA = 6, with the Kinship for MLM, CMLM, MLMM, SUPER; and Pseudo QTNs for FarmCPU and BLINK.

### SNP selection accuracy and selection efficiency

The accuracy and efficiency of SNP selection were computed to evaluate the significant SNP by the allele proportion in the population.

*Selection accuracy* (SA) = 100*[(Number of S or R genotypes with the favorable SNP allele)/(Number of R genotypes with the favorable SNP allele + Number of S genotypes with the favorable SNP allele)]/ΔE, where ΔE = E_1_/E_2_, E_1_ = Observed number of S or R genotypes/(Number of R genotypes + Number of S genotypes); E_2_= Expected number of S or R genotypes/(Number of R genotypes + Number of S genotypes).

*Selection efficiency* (SE) =100*[(Number of S or R genotypes with the favorable SNP allele)/(Total number of S or R genotypes)]/ΔF, where ΔF = F_1_/F_2_, F_1_ = Observed allele frequency of SNP, and F_2_ = Expected allele frequency of SNP. In this study we set the E_2_ and F_2_ as an ideal equilibrium value (50%).

### Candidate gene prediction

Candidate genes were selected based on the peak significant SNP in each LD region located within 50 kb on either side of significant SNPs (Zhang et al., 2016), and furtherly by 0 kb (on the gene), 1 kb, 5 kb, 10 kb, 20 kb, 30 kb, and 50 kb, respectively. Candidate genes were retrieved from the reference annotation of the soybean reference genome Wm82.a2.v1 from the SoyBase (http://www.soybase.org) and the Phytozome database (https://phytozome.jgi.doe.gov).

### Genomic prediction

GP was conducted using seven SNP sets: All SNP set (30,314 SNPs) and six GWAS-derived SNP marker sets. The six GWAS-derived SNP marker sets consisted of those significant SNPs from highest LOD [–log(P-value)] to low LOD value (2.0) to make each set as 28, 100, 500, 1,000, 2,000, and 5,000 SNPs, respectively. Genomic estimated breeding value (GEBV) was computed using five statistical models: Ridge regression best linear unbiased predictor (rrBLUP) (Endelman, 2011), Genomic best linear unbiased predictor (gBLUP) (Zhang et al., 2007), Bayesian least absolute shrinkage and selection operator (Bayesian LASSO) (Heslot et al., 2012), Random Forest (RF) (Poland et al., 2012), and Support vector machines (SVM) (Ogutu et al., 2011) (**Table S2**).

A five-fold cross-validation was performed for each GP. The association panel was randomly divided into 5 disjoint subsets, 4 subsets were used as training set, and the remaining set was considered testing set. A total of 100 replications were conducted at each fold. Mean and standard errors corresponding to each fold were computed. Genomic prediction accuracy was obtained by computing the Pearson’s correlation coefficient (r) between GEBV and the observed phenotypic value for the testing set (Shikha et al., 2017).

## Results

### Germplasm evaluation of *Phakopsora pachyrhizi*

**Figure 1:**
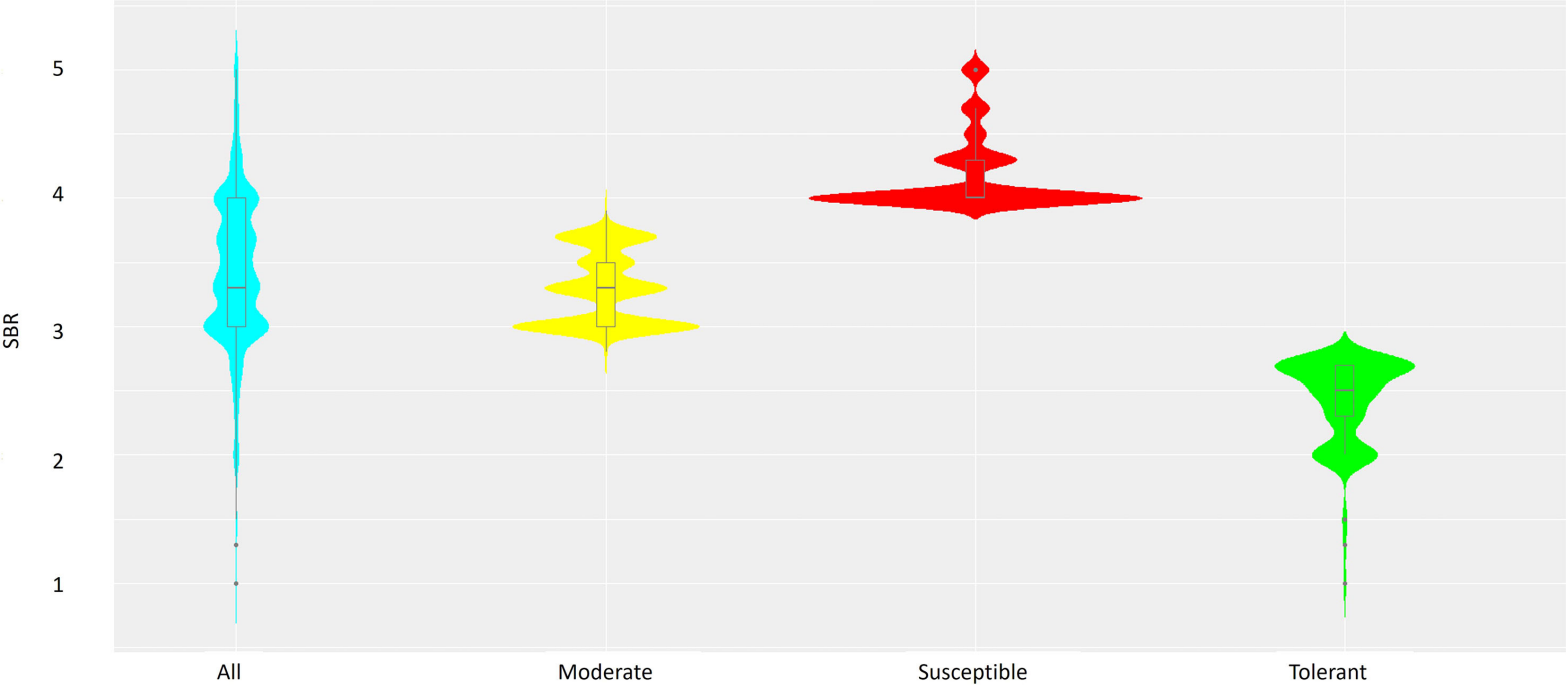
Combined violin-boxplots based on SBR ranking of the 3,082 soybean accessions, including Susceptible (red), Moderate (yellow) and Tolerant (green) groups.

Out of 3,082 soybean accessions evaluated for TAN lesion type, 71 (2.3%) were rated 1~2, 1,009 (32.7%) were rated 2.3~3, 1,746 (56.7%) were rated 3.1~4; and 256 (8.3%) were rated 4.2~5 in a rating scale of 1 to 5. Accessions with a mean severity of 2.7 or less (299, 9.5%) were considered resistant, while those with a mean severity of 4.0 or more (791, 25.6%) were considered susceptible. Accessions between the two categories were considered moderate. There was a large range in the distribution of each category (**Figure 1**). Majority of accessions displayed a disease severity rating of 3 or 4 being susceptible to SBR.

### SNP profile

**Figure 2:**
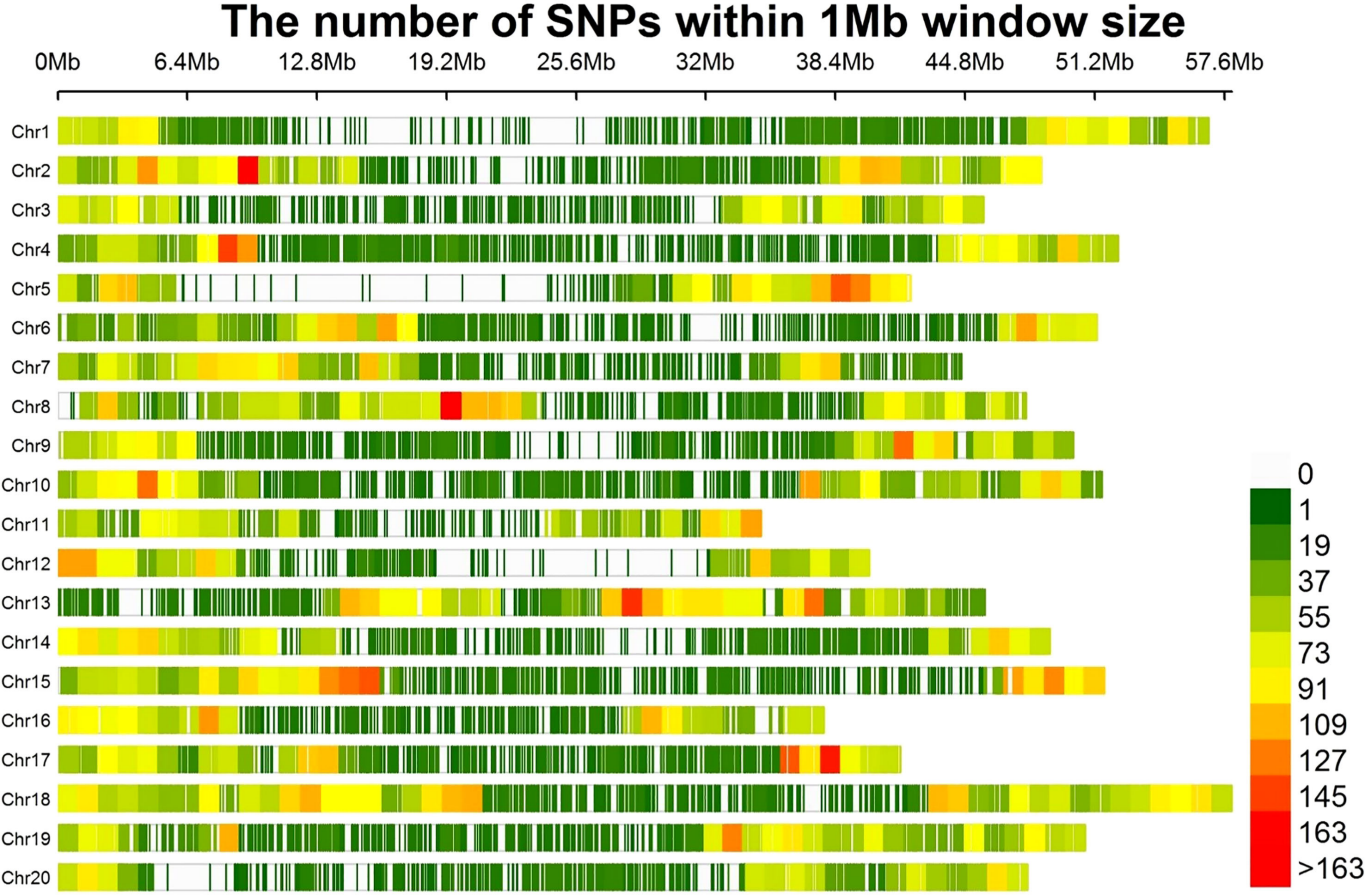
The distribution of 30,314 SNPs among the 20 chromosomes of soybean within 1 Mb size.

A total of 30,314 high quality SNPs were used to perform GWAS in the soybean accessions. Number of SNPs per chromosome ranged from 1,027 on chr20 to 1,898 on Chr16, with an average of 1,515.7 SNPs (**Figure 2**). The average distance between two SNPs per chromosome varied from 23.6 kb to 46.6 kb, with an average of 33.1 kb. The shortest average distance between SNPs was found on Chr18, whereas the longest one was on Chr20. Average MAF per chromosome ranged between 25.8% and 30.1%, with an average of 28.7% (**Table S3**). Percentage of heterozygous SNPs across all chromosomes were below 0.7%, and the percentage of missing SNPs per chromosome varied from 0.3% to 0.7%.

### Population structure and LD haplotype

**Figure 3:**
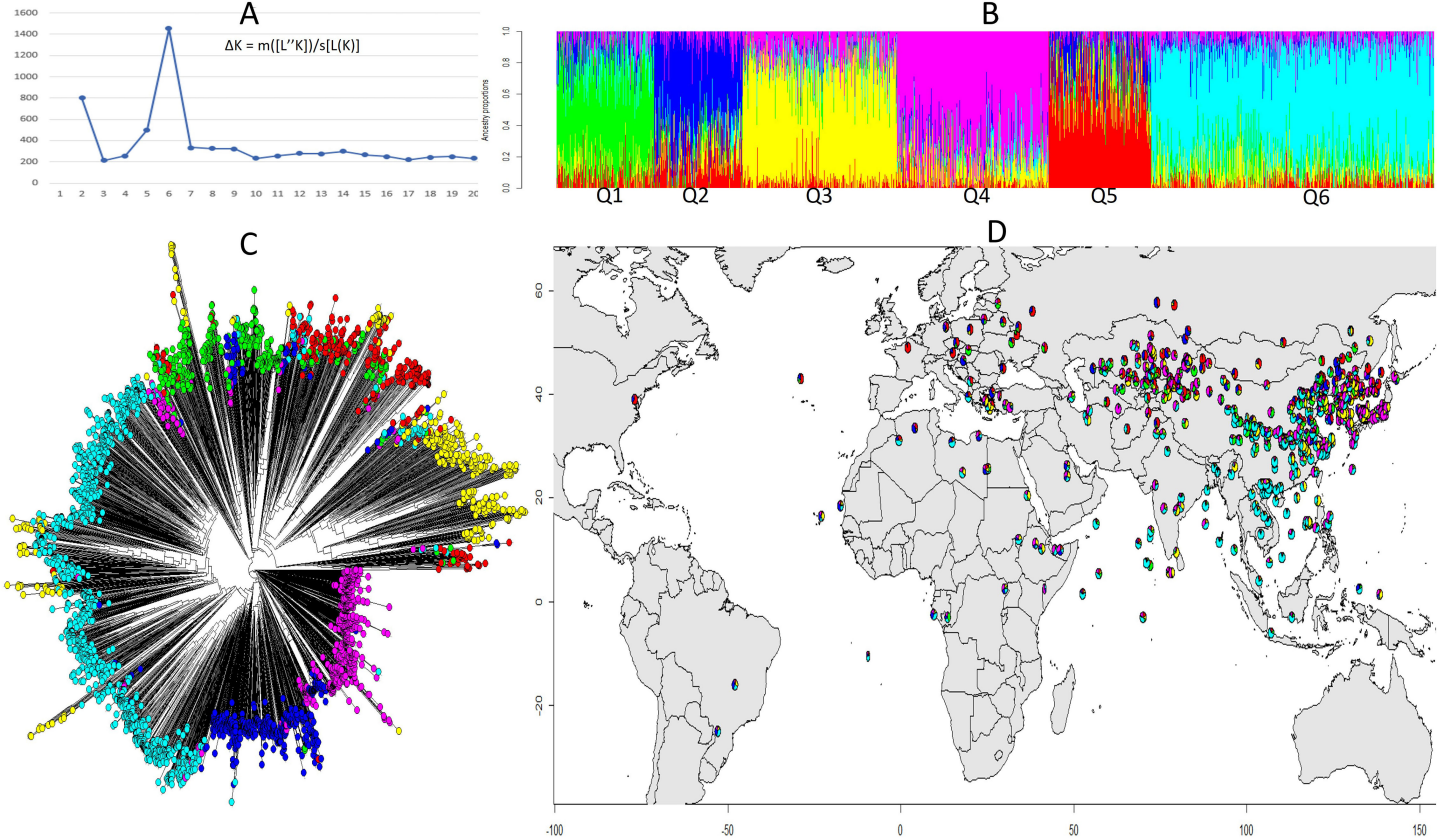
Structural and phylogenetic analysis of 3,082 soybean accessions based on 30,314 SNPs. **(A)** Delta K values for different numbers of populations assumed (K=20) in the STRUCTURE analysis. **(B)** Classification of soybean accessions in six groups (K=6) using STRUCTURE. The distribution of accessions to different populations is color coded, Q1 (green), Q2 (blue), Q3 (yellow), Q4 (pink), Q5 (red), Q6 (cyan). The x-axis shown the accessions of each subgroup, and the number on the y-axis shows the Q likelihood of accessions. **(C)** Phylogenetic analysis of the 3,082 soybean accessions with the corresponded labels used in **(B)**. **(D)** Geographical distribution of the soybean accessions by colored pie chart corresponding with the group proportion **(B)**.

The structure analysis helped identify the most promising genetic variations to better understand the genetic basis of the trait. The population structure of the soybean accessions was analyzed using the R packages “LEA” and the peak of ΔK was observed at K = 6, indicating of the presence of six subpopulations or clusters (**Figure 3A**). A total of 337 (10.9%) accessions were assigned to subpopulation Q1; 306 (9.9%) assigned to Q2; 543 (17.6%) assigned to Q3; 534 (17.3%) assigned to Q4; 358 assigned to Q5; and 1,004 (32.5%) assigned to Q6 (**Figure 3B**). Phylogenetic analysis and population admixture map using R packages “Phytool” and “LEA” also revealed that the clustering of accessions was consistent with that inferred by structure K = 6 (**Figure 3C**). Additionally, there was a clear tendency of clustering by geographical areas. The controlling for population structure by taking geography into account is crucial for accurate GWAS results and for identifying true genetic associations with the trait of interest. As Q6 was dominant in South and Central China and Southeast Asia, Q3 and Q4 were main populations in Northeast and Northwest Asia, and the population in Europe was dominated by Q2 and Q5 (**Figure 3D** and **Table S1**). Kinship matrix, based on 30,314 polymorphic SNPs for the studied genotypes, indicated that there was no clear clustering among the 3082 genotypes.

**Figure 4:**
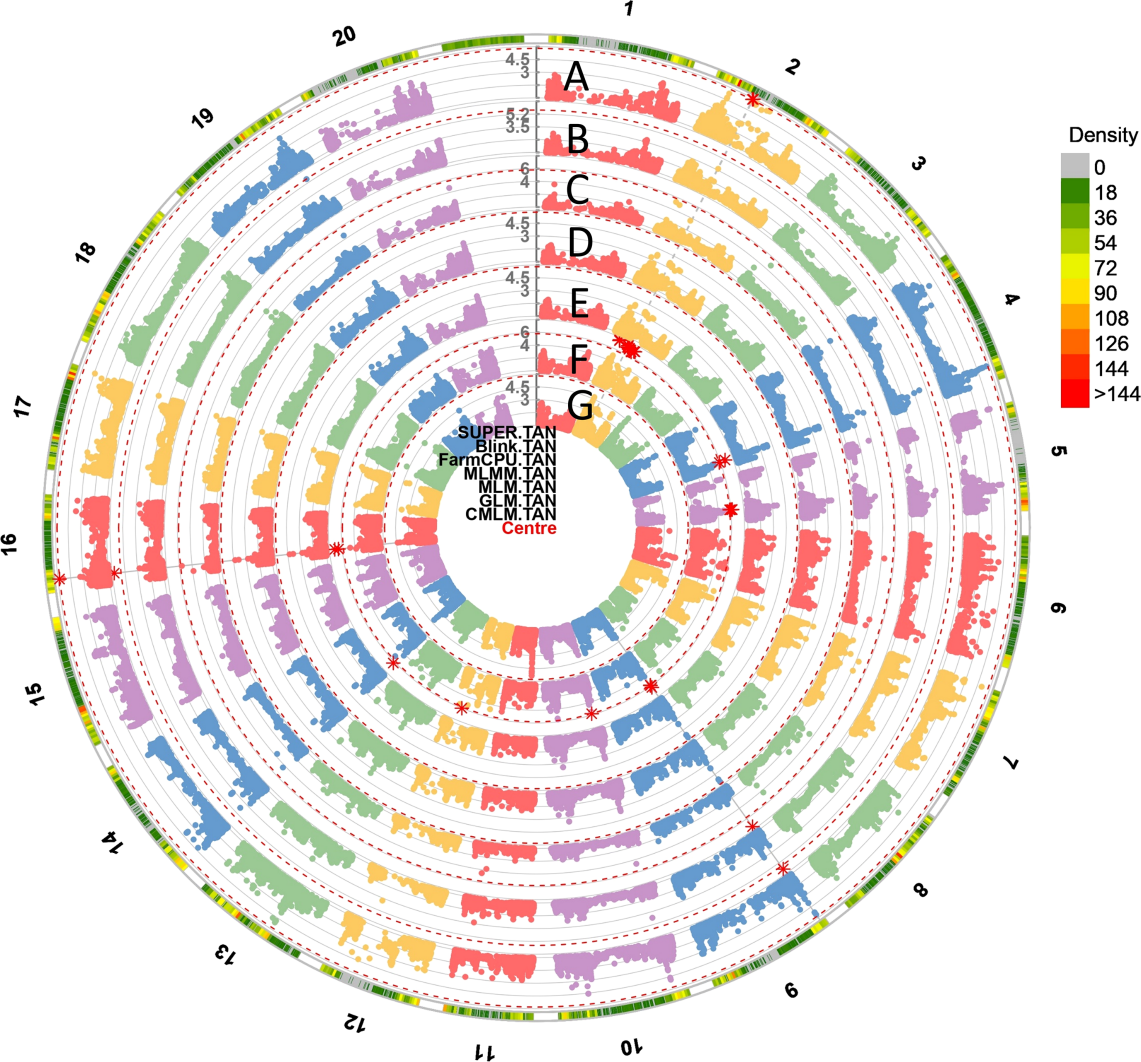
The circular Manhattan plots of seven GWAS models: **(A)** Settlement of MLM Under Progressively Exclusive Relationship (SUPER), **(B)** Bayesian-information and Linkage-disequilibrium Iteratively Nested Keyway (BLINK), **(C)** Fixed and Random Model Circulating Probability Unification (FarmCPU), **(D)** Multiple Loci Mixed Model (MLMM), **(E)** Mixed Linear Model (MLM), **(F)** Generalized Linear Models (GLM) and **(G)** Compressed Mixed Linear Model (CMLM) for SBR. The red asterisk points to the significant spots associated with SBR on 20 chromosomes. The outmost circle indicates the hotspots associated with SBR response among seven models.

We examined the linkage disequilibrium (LD) decay patterns by 30,314 genome-wide SNPs. To visualize the LD decay patterns across distances, we plotted the LD decay curves by GAPIT 3 (**Figure 4**). The LD decay curves showed a clear distance-dependent pattern, with steeper decay curves at longer distances. Specifically, at a distance of 103 kb, the LD decayed with an R^2^ value of 0.6, indicating a relatively strong LD correlation between nearby variants. At 216 kb, the LD decayed with an R^2^ value of 0.4, indicating a moderate level of LD correlation between nearby variants. Finally, at 296 kb, the LD decayed with an R^2^ value of 0.2, indicating a weak level of LD correlation between nearby variants (**Figure S1**). A total of 4,940 haplotype blocks were identified based on 30,314 SNPs. Number of blocks per chromosome varied from 170 on Chr11 to 357 on Chr18. Number of SNPs within each block varied from 2 to 67. Many haplotype blocks contained more than two significant SNP markers, for example, Gm01_47,462,126, Gm01_47,476,910, Gm01_47,481,216, Gm01_47,495,955, Gm01_47,503,665, Gm01_47,516,500, and Gm01_47,548,257 were in the same haplotype block on Chr1 (**Table S4**).

### Genome-wide association study

The high convergence and consistency of the GWAS were observed among seven models. For example, the top six significant SNPs from the FarmCPU model including: Gm09_1,951,644 (10.06), Gm20_36,724,867 (6.54), Gm03_38,913,029 (6.10), Gm19_44,734,953 (5.7), Gm02_7,235,181 (5.18), and Gm04_47,132,429 (5.06) also had the high LOD values, which were at least 5.20, 2.67, 3.77, 3.59, 3.69, and 4.00 in other models. SNPs Gm04_45,884,688, Gm10_39,142,024, Gm14_2,492,139, Gm16_4,935,328, etc. were significant among all seven models (**Figures 4**, **S2**). A total of 100 SNPs were collected in this study by considering both model consistency and significance (**Table S5**). These SNPs were positioned at 47 haplotype blocks (**Table S4**). Then the top 28 SNPs with LOD > 5.50 were listed in **Table 1** for future discussion. These 28 SNPs were located on 13 chromosomes (Chr. 2, 3, 4, 6, 8, 9, 10, 12, 13, 14, 16, 19, and 20), indicating their wide distribution and presence of genes that confer SBR resistance across the genome. Several SNPs were found in the same blocks, such as Gm02_7,235,181 and Gm02_7,234,594 in block 436; Gm09_1,944,730, Gm09_1,943,831, and Gm09_1,951,644 in block 1902; Gm10_5,573,877, Gm10_5,573,007, Gm10_5,559,592, Gm10_5,541,691, and Gm10_5,578,693 in block 2331; and Gm10_39,142,024 and Gm10_3,9147,121 in block 2215, which might be due to the gene clustering or pleiotropy.

**Table 1:** The genes within 50 kb genomic region of the top 28 significant SBR-associated SNPs with functional annotations.

SNP	GWAS model (Ranking)	LOD	Allele Type	Gene name	Functional annotations
Gm02_7235181	SUPER(1), FarmCPU, CMLM(5), MLMM(10)	7.91	T/C	Glyma.02G083500 Glyma.02G083300 Glyma.02G084100	LRR; RCC1; response to bacterial origin; defense response; structural constituent of cell wall
Gm02_7234594	SUPER(2), MLMM(11)	7.61	C/T		
Gm02_7315227	SUPER(3), GLM,MLM, Blink(5), MLMM(6)	7.52	G/A	Glyma.02G084100 Glyma.02G084900	RCC1 repeat; Ankyrin repeat family protein/domain
Gm03_38913029	GLM, MLM, Blink (2), FarmCPU, CMLM(3), MLMM(7), GLM	6.85	T/C	Glyma.03G175800 Glyma.03G177400 Glyma.03G175300	Response to aluminum ion; cell wall; ABC transporter
Gm04_45884688	MLM, Blink(7), SUPER(15), MLMM(16), FarmCPU, CMLM(26)	6.23	T/C	Glyma.04g188000	LRR
Gm04_46003059	SUPER(20), MLMM(24)	6.03	G/A	Glyma.04G189300, Glyma.04g189500	Membrane; Cytochrome P450
Gm04_46295839	SUPER(16), MLMM(18)	6.08	C/T	Glyma.04G192300	Cell wall organization; cellular membrane fusion;
Gm04_46389651	SUPER(22), MLMM(27)	5.94	C/T		
Gm04_47132429	MLMM(4), FarmCPU, CMLM(6), GLM, MLM, Blink(13), SUPER(25)	5.78	T/C	Glyma.04G211100, Glyma.04G212000	NAC domain
Gm06_36808946	SUPER(6), GLM, MLM, Blink(9), FarmCPU, CMLM(34)	6.73	G/A	Glyma.06G232500	Response to molecule of bacterial origin
Gm08_43955878	FarmCPU, CMLM(19), SUPER(32), MLMM(33)	5.61	A/C	Glyma.08g319300, Glyma.08G321700	LRR; response to abscisic acid stimulus/cold/water deprivation
Gm09_1944730	MLMM(2), SUPER(27)	5.77	C/A	Glyma.09G024700	LRR-RLKs
Gm09_1943831	MLMM(3), SUPER(28)	5.73	G/A		
Gm09_1951644	FarmCPU, CMLM, MLMM (1), GLM, MLM, Blink (4),SUPER(18)	10.07	T/G		
Gm10_5573877	SUPER(5), MLMM(12), GLM, MLM, Blink(14)	6.73	C/T	Glyma.10G060100, Glyma.10G060200, Glyma.10G060600	Respiratory burst involved in defense response, response to bacterium/chitin; cell wall organization
Gm10_5573007	SUPER(7), MLMM(15)	6.58	C/T		
Gm10_5559592	SUPER(9), MLMM(20)	6.48	C/A		
Gm10_5541691	SUPER(33), MLMM(44)	5.60	C/T		
Gm10_5578693	SUPER(23), MLMM(32)	5.93	G/A		
Gm10_39142024	GLM, MLM, Blink(1), MLMM(8), SUPER(10), FarmCPU, CMLM(14)	7.12	C/T	Glyma.10g157500	LRR-RLKs, regulation of plant immunity
Gm10_39147121	MLMM(9), SUPER(21)	6.02	T/G		
Gm12_28136735	SUPER(4),GLM,MLM, Blink(8), MLMM(39)	7.03	G/A	Glyma.12G160100, Glyma.12G160400	NAC domain protein; Cytochrome P450
Gm13_16350701	FarmCPU, CMLM(16), GLM, MLM, Blink(23), SUPER(29)	5.63	T/C	Glyma.13G064500	F-box and WD40 domain protein, disease resistance protein
Gm14_2492139	GLM, MLM, Blink(6), SUPER(13), FarmCPU, CMLM(25), MLMM(26)	6.26	A/C	Glyma.14G034200, Glyma.14G040000	RCC1 family protein; LRR-RLKs
Gm14_6185611	MLMM(28), SUPER(36), GLM, MLM, Blink(46)	5.51	C/T	Glyma.14g073300, Glyma.14G073800	F-box domain; regulation of defense response
Gm16_4935328	GLM, MLM, Blink(10), MLMM(22), SUPER(31), FarmCPU, CMLM(32)	5.61	T/G	Glyma.16G051800, Glyma.16G052200	NAC domain protein; LRR-RLKs
Gm19_44734953	GLM, MLM, Blink(3), FarmCPU, CMLM(4), MLMM(25)	6.02	G/A	Glyma.19G189900, Glyma.19G190200, Glyma.19G190800	Defense response to bacterium; LRR-RLKs; plant-type cell wall
Gm20_36724867	FarmCPU, CMLM(2)	6.54	C/T	Glyma.20G124700	QSOX1 regulates plant immunity

### Candidate genes of significant SNPs

Due to variations in LD decay across different regions, a conservative distance of 50 kb was set to select candidate genes as the region of the significant SNPs. There are four SNPs (loci) out of the top 100 associated markers, including Gm18_57,223,391, Gm16_29,491,946, Gm06_45,035,185, and Gm18_51,994,200, were identified to locate in close proximity to four main *P. pachyrhizi* R genes Rpp1, 2, 3, and 4, respectively, which were verified and reported in last decades.

Thirty-five candidate genes that might be associated with SBR disease resistance were found in the regions of the top 28 significant SNP markers (**Table 1**). Disease related annotations of these candidate genes were included but not limited to: LRR (Leucine Rich Repeat class protein), cytochrome 450, cell wall structure, RCC1 (regulator of chromosome condensation 1), AKR (ankyrin repeat-containing protein), F-box domain, NAC (NAM, ATAF and CUC family). Furthermore, most of the top 28 significant SNP regions were harboring more than one candidate gene, for example, the region of Gm02_7,235,181 and Gm02_7,234,594 contained three candidate genes, Glyma.02G083500, Glyma.02G083300, and Glyma.02G084100, coding for cell wall constituent, LRR-RLK, and RCC1, respectively.

### Selection accuracy and selection efficiency

Selection accuracy (SA) and Selection efficiency (SE) reflect the contributions of selected alleles from the top 100 significant SNP to the resistance or susceptibility to *Phakopsora*. For the resistance alleles, SE varied from 50.0% to 84.2%, with an average of 57.5%; and the SA varied from 50.0% to 82.2%, with an average of 58.2%. SNP Gm09_1,951,644 had the highest values in both SA and SE in resistance effect. For susceptible alleles, the SE varied from 50.0% to 69.8%, with an average of 55.1%; and the SA varied from 50.3% to 56.9%, with an average of 52.7%. SNP Gm04_46,295,839 (52.7%) had the highest values in both SA and SE in susceptible effect (**Table S6**). This result identified the specific nucleotide of SBR-related alleles.

### Genomic prediction

**Figure 5:**
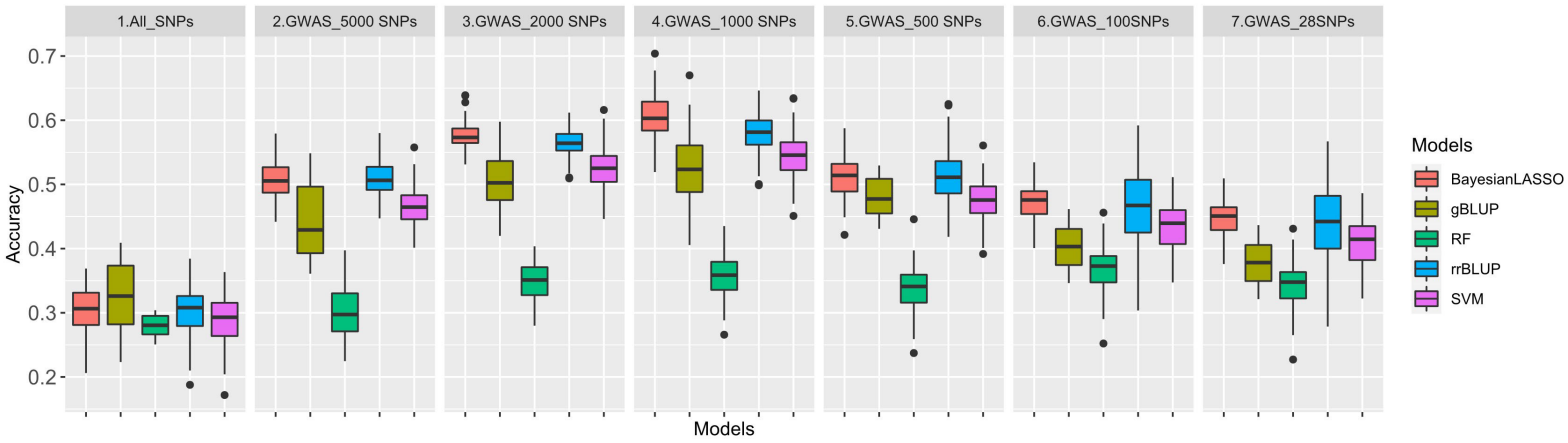
Genomic prediction (GP) accuracy for rust using five GP models: Ridge regression best linear unbiased predictor (rrBLUP) = blue, Genomic best linear unbiased predictor (gBLUP) = dark yellow, Bayesian least absolute shrinkage and selection operator(Bayesian LASSO) = red, Random Forest (RF) = green, Support vector machines (SVM) = purple based on seven datasets: All_SNPs (30314), and six GWAS based SNP sets with top28, 100, 500, 1,000, 2,000 and 5,000 SNPs.

The 100 significant SNPs not only had the highest LOD value but were most repeatable across all GWAS methods as well. Following the same approach, six additional GWAS-based SNP sets were created, each consisting of 28, 100, 500, 1,000, 2,000, and 5,000 SNPs, respectively. In this study, we applied seven datasets, namely, All_SNPs (30,314), GWAS_5000SNPs, GWAS_2000SNPs, GWAS_1000SNPs, GWAS_500SNPs, GWAS_100SNPs and GWAS_28SNPs for GP analysis by five different GS models (**Figure 5**). The average GS accuracies of the All_SNPs set were at a medium level that was similar to those, ranging from 28.0% (RF) to 32.4% (gBLUP), among all the models.

Although the number of SNPs fluctuated by GWAS datasets, all the accuracy curves showed a similar pattern among the five models. The trend depicted by the left side of the curves indicated that as the number of SNPs decreases from 5,000 to 1,000, the accuracy of the prediction increases, too. The highest accuracies were observed when using the 1,000 SNP set, which were varying from 35.7% (RF) to 60.4% (Bayesian LASSO). And, as the number of SNPs continued to decrease from 1,000 to 100, the accuracy of GP also decreased. In all six GWAS based SNP sets, the Bayesian LASSO achieved the highest average GS accuracy of 53.0%, followed by rrBLUP with an average accuracy of 51.9%. On the other hand, the lowest accuracy of 36.2% was recorded when using the RF model. The GS accuracies of gBLUP and SVM models were at almost the same level but were relatively lower using the SVM model (**Table S7**).

## Discussion

### Phenotype

Resistance to *P. pachyrhizi* is commonly evaluated based on three types of SBR lesions: “TAN”, “RB”, and “Mixed”. The “TAN” lesion type is characterized by heavy fungal sporulation typically develop on susceptible soybean leaves, while the RB or “reddish-brown” lesion type has been linked to resistance in known single gene resistance. The “Mixed” reaction is recorded when both RB and TAN lesions were observed on the same leaf (Miles et al., 2006). The simple classification of TAN and RB lesions had been widely used decades ago; however, it had been noted as oversimplified to the symptom observation. Nowadays, the appropriate practice is to separately divide TAN and RB into multiple classes to provide more accurate descriptions of disease symptoms while taking into account variations in fungal sporulation. Considering data consistency and method popularity, we took the TAN lesion as the phenotype of the association analysis for this study, which had sufficient observations and good distribution of SBR resistance. In the present study, the resistance resources were primarily sourced from China, Japan, and Korea, comprising 40%, 16%, and 21% of the total resources, respectively. These figures closely align with the respective proportions of 43%, 13%, and 18% observed in the overall population. In addition, according to the ANOVA between groups, it is obvious that the variability (99%) within groups is greater than the variability (1%) between groups (**Table S8**).

### GWAS and candidate genes

Specific resistance to *P. pachyrhizi* is controlled by seven single dominant genes, namely, *Rpp1* (Chr 18), *Rpp2* (Chr16), *Rpp3* (Chr6), *Rpp4* (Chr7), *Rpp5* (Chr3), *Rpp6* (Chr18), and *Rpp7* (Chr19) (Calvo et al., 2008; Meyer et al., 2009; Lemos et al., 2011; Childs et al., 2018b). The single genes played an important role in SBR resistance, but this kind of resistance is not durable, and the usefulness of the sources loses its effectiveness once it is identified and applied in breeding (Chander et al., 2019). GWAS was performed in efforts to discover loci contributing SBR resistance, thus helping find all genes for SBR control (Chang et al., 2016). Multiple models were developed for GWAS based on linkage disequilibrium, including GLM, MLM, CMLM, MLMM, SUPER, FarmCPU, and BLINK (Wang and Zhang, 2021). Previous studies demonstrated that the differences of the models were caused by the interactions between the methods and other factors, including populations, sample size, mapping resolution, trait complexity, and quality of the data. Typically, all GWAS methods perform well when the aforementioned factors are favorable; however, each model may have varying numbers of false positives depends on the strengths and weaknesses of the model in different circumstance. Therefore, it is important to carefully consider the advantages and limitations of each GWAS method and choose the most appropriate one for the specific study and data. Additionally, multiple methods and independent replication studies are often used to confirm the validity of the results and minimize the risk of false positive findings. However, GWAS studies on SBR resistance were scarce, with the exception of a few studies that used a single model to discover loci contributing to general disease resistance in soybean (Kang et al., 2012; Chang et al., 2016). In this study, we applied all seven models and also considered both significance and consistency of each model for candidate SNPs of SBR resistance to hedge the false positives.

A total of four significant SNPs were located on or nearby the reported R genes. SNP Gm06_45,035,185 in chromosome 6 was located at gene *Rpp3*; Gm18_51,994,200 and Gm18_57,223,391 in chromosome 18 were nearby the genes *Rpp4*/*Rpp4-b* and *Rpp1*/*Rpp1-b*, respectively; and Gm16_29,491,946 in chromosome 16 was located at *Rpp2*, which showed the promise of GWAS on SBR resistance (Sharma and Gupta, 2006). However, we only observed moderate significance for these four SNPs in GWAS analysis, probably due to the following reasons: 1) different genetic variants contributing to the trait, rather than a single major gene; 2) major genes are often rare, the signal from a major gene may be diluted by underrepresented or even missing gene(s) in the samples.

Except for the major *Rpp*s, some significant SNPs also associate with *LRR* class genes that were considered to be the majority of disease resistance genes in plants (Kang et al., 2012). Genes encoding cytochrome P450 have been shown to contribute to both plant development and defense under pathogen attack (Siminszky et al., 1999). The F-box family proteins have been demonstrated to be directly involved in plant defense against pathogens(Liu and Xue, 2011). The QSOX1 (quiescin sulfhydryl oxidase homolog) were reported to negatively regulate plant immunity against a pathogen (Chae et al., 2021); WD40 repeat-containing proteins which played an important effect on plant defense (Miller et al., 2016). The results were indicative of the robustness of the significant SNPs identified in this study. Other functional annotations pertaining to the candidate genes of cell wall structure/organization/construction and membrane fusion/proteins/structure/transporters have been demonstrated to play some roles in plant passive defense to pathogens (Mellersh and Heath, 2001; Hématy et al., 2009). The RCC1, NAC domain protein, ABC (ATP-binding cassette) transporters, etc. involve in many plant response-associated physiological activities to biotic or abiotic stresses and are widely annotated to the candidate genes (**Table 1**, **S5**) (Langenbach et al., 2016; Gautam et al., 2020; Oh et al., 2022). Furthermore, previous studies have reported the involvement of LRR (leucine-rich repeat), ABC transporters and F-box proteins in conferring resistance to rust fungi in other crop species belonging to the same order of *Pucciniales*, including wheat (Vikas et al., 2022), barley (Sallam et al., 2017), and maize (Juliana et al., 2018).

### Selection accuracy and selection efficiency

SE and SA were computed for the significant SNPs associated SBR resistance or susceptibility (Ravelombola et al., 2017). The SA and SE of the marker were measured by relative proportion of an allele type (A/T/C/G) in resistant or/and susceptible accessions, as has been highlighted in other GWAS-related reports (Shi et al., 2016; Ravelombola et al., 2019). Specifically, the proportion of allele type for a completely un-associated SNP should be close to 50% in either resistant or susceptible group. Therefore, when the SA or SE value of the allele type is more than 50%, it contributes positively to the corresponding trait, or vice versa. In general, the two different nucleotides of any significant SNP must have opposite effects on disease resistance or susceptibility, which are defined as “R” or “S” alleles. We observed significant difference between “R” and “S” alleles in one SNP. For example, the “R” allele of SNP Gm04_46,295,839(C/T) has a “C” nucleotide with low SE and SA values (52.6% and 53.9%), but its “S” allele has a “T” nucleotide with high SE and SA values (67.8% and 57.%). This locus may relate to a S gene encoding a cellular membrane fusion protein as annotated in this study. On the contrary, the “A” allele of SNP Gm08_46,674,632(G/A) has high SE (84.2%) and SA (82.2%) values with resistance effect, whereas its “G” allele has low SE (51.5%) and SA (51.4%) values with susceptible effect. This locus is more likely to associate with a R gene coding for a LRR-containing protein in this study. In this study, all significant SNPs have higher than expected SA and SE values (>50%), suggesting that these SNPs can be used for further marker-assisted selection to enhance SBR resistance breeding in soybean.

### Genomic prediction

The study discovered 28 significant SNPs located in 20 loci with genes that are associated with plant disease response or resistance. However, before applying these findings in breeding, further verification work is needed (Jannink et al., 2010; Crossa et al., 2017). GS has gained popularity in recent years in large-scale crop breeding programs. Previous studies have shown that GS achieves a more robust prediction of genotypic values compared to QTLs for traits controlled by many genes with small effects. GS tends to have a better and more reliable prediction than the traditional QTL approach, because it uses more markers that are distributed throughout the genome and captures more genetic variation of a trait (Bhat et al., 2016). GS can make predictions about an individual’s performance even before it is phenotyped, which can save time and resources in the breeding process (Zhang et al., 2016; Ravelombola et al., 2019).

However, no research has investigated GS or GP for SBR resistance/tolerance. In this study, we performed GP with seven models on one All_SNP set and six GWAS-based SNP sets. The accuracies of All_SNP set (28.0%~32.4%) were similar to former studies on resistance/tolerance traits to abiotic and biotic stresses of several plant species, including wheat (Poland and Rutkoski, 2016), rice (Xu, 2013), maize (Technow et al., 2013), canola (Jan et al., 2016), alfalfa (Hawkins and Yu, 2018), cassava (Ly et al., 2013), oats (Asoro et al., 2011), miscanthus (Olatoye et al., 2020), grapevine (Brault et al., 2022), and intermediate wheatgrass (Crain et al., 2020). On the other hand, GWAS_SNPs-based GP accuracies were higher than those of All_SNP set-based, demonstrating the importance and contribution of significant SNPs in SBR resistance/tolerance. The accuracy using linear model gBLUP (45.5% in average) was close to those from machine learning (SVM), 47.5% in average, but lower than rrBLUP (51.2% in average) and Bayesian LASSO (52.0% in average) that had been considered to be the optimal approaches (Ravelombola et al., 2019).

Consistently with former reports (Bao et al., 2014; Li et al., 2018), we observed in this study that the accuracy of GP varied by the number of SNPs. For those GWAS-based SNP sets, a greater proportion of SNPs with more significance were retained for GS after further filtering of markers from 5,000 to 1,000, which led to increased accuracy. The accuracies of all models were improved until the number of SNPs reached 1,000, after which the accuracies began to decline until the number of markers dropped to 28. The apex of predictive accuracy was observed at a SNP count of 1,000, likely due to its ability to robustly capture LD and account for relatedness among soybean genotypes. An excess of SNPs beyond this threshold would introduce extraneous information to the models and elevate model complexity, while a SNP count lower than 1,000 would result in the loss of relevant information regarding LD and relatedness capture. Then again, the objective of this GWAS study was primarily to identify the associated loci and candidate genes related to SBR. The use of multiple SNP sets and GS models was employed to ensure the consistency of the GWAS results, rather than to quantitatively evaluate the superiority or variations between the models and data sets. However, the above results can still serve as a reference for future GS research in disease resistance.

Phenotypic selection has been successfully implemented for disease resistance, but without controlled experiments, it is difficult to determine whether the resistance is quantitative or qualitative. Therefore, it is difficult to determine whether the resistance will be durable in the long term. In this study, the SBR-related markers we identified can be used to select for both quantitative and qualitative disease resistance within the breeding lines to bypass the need for controlled experiments through the use of conventional MAS. Additionally, by utilizing GP, the breeders can select for the accumulation of QTL associated with resistance, thereby taking advantage of both quantitative and qualitative resistance genes, even those that have not yet been characterized. This allows them to select the most promising lines for further development and testing without multiple generations of phenotyping.

## Data availability statement

The original contributions presented in the study are included in the article/**Supplementary Material**. Further inquiries can be directed to the corresponding authors.

## Author contributions

HX, AS, JW, and YC organized and analysed the original data. HX and Y-BP drafted the manuscript. WL, Y-BP and AS critically revised the manuscript. All authors contributed to the article and approved the submitted version.

## Supplementary material

Supplementary Figure 1 The linkage disequilibrium decay rate was estimated as squared correlation coefficient (r^2^) using all pairs of SNPs located within 4 Mb of physical distance in euchromatic. The red line is the moving average of the (r2) value of the ten adjacent markers.

Supplementary Figure 2 The Manhattan plots for SBR by multi-GWAS models: **(A)** Blink, **(B)** GLM, **(C)** MLM, **(D)** CMLM, **(E)** FarmCPU, **(F)** SUPER, **(G)** MLMM. Additionally: **(H)** QQ-plots of the above seven models.
