# Supplementary figures and images for: A genome-wide association study and genomic prediction for Phakopsora pachyrhizi resistance in soybean
Source: Front Plant Sci. 2023 May 29;14:1179357. doi: 10.3389/fpls.2023.1179357 (PMC10258334; doi:10.3389/fpls.2023.1179357)

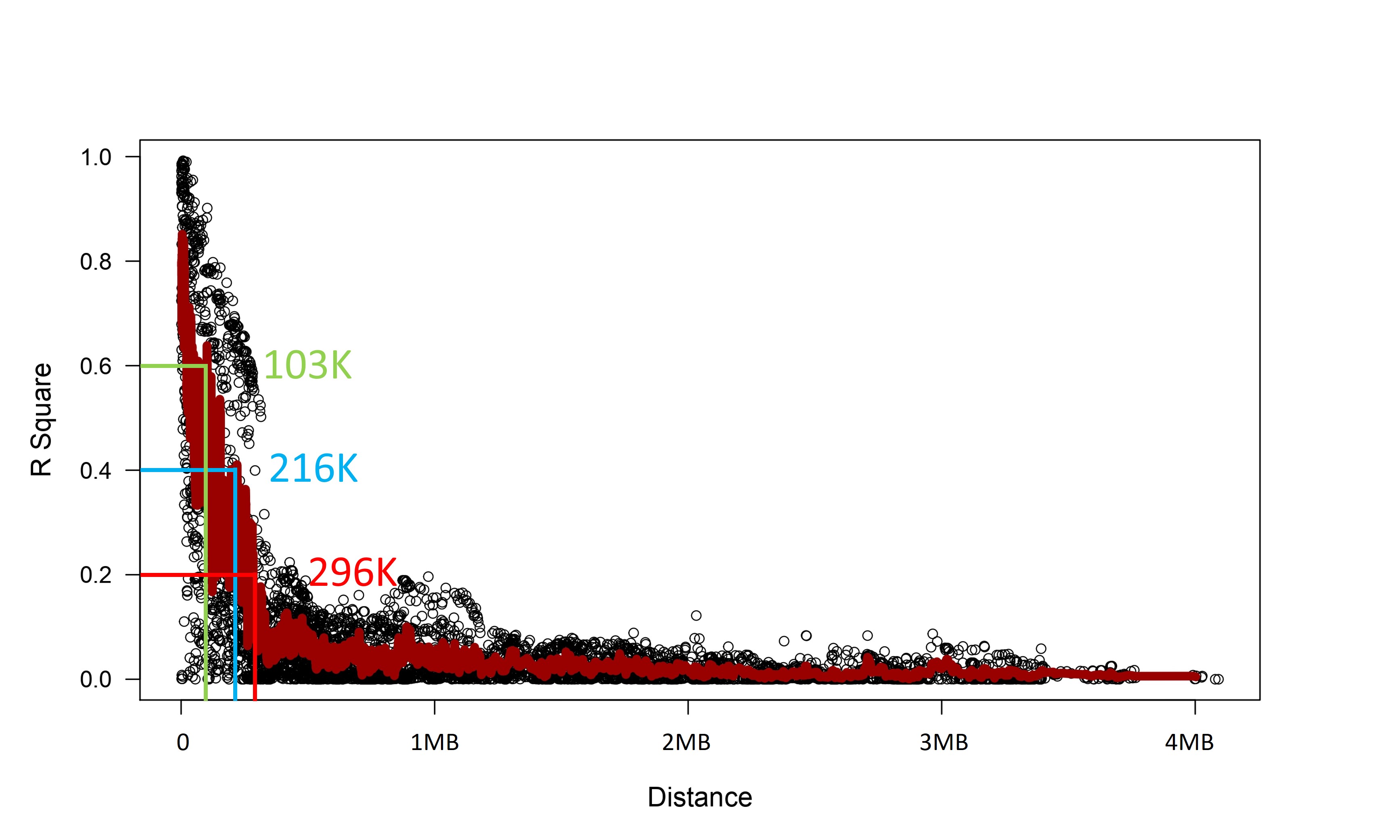

Supplement: Supplementary Figure 1 — The linkage disequilibrium decay rate was estimated as squared correlation coefficient (r2) using all pairs of SNPs located within 4 Mb of physical distance in euchromatic. The red line is the moving average of the (r2) value of the ten adjacent markers. [file Image_1.jpeg]

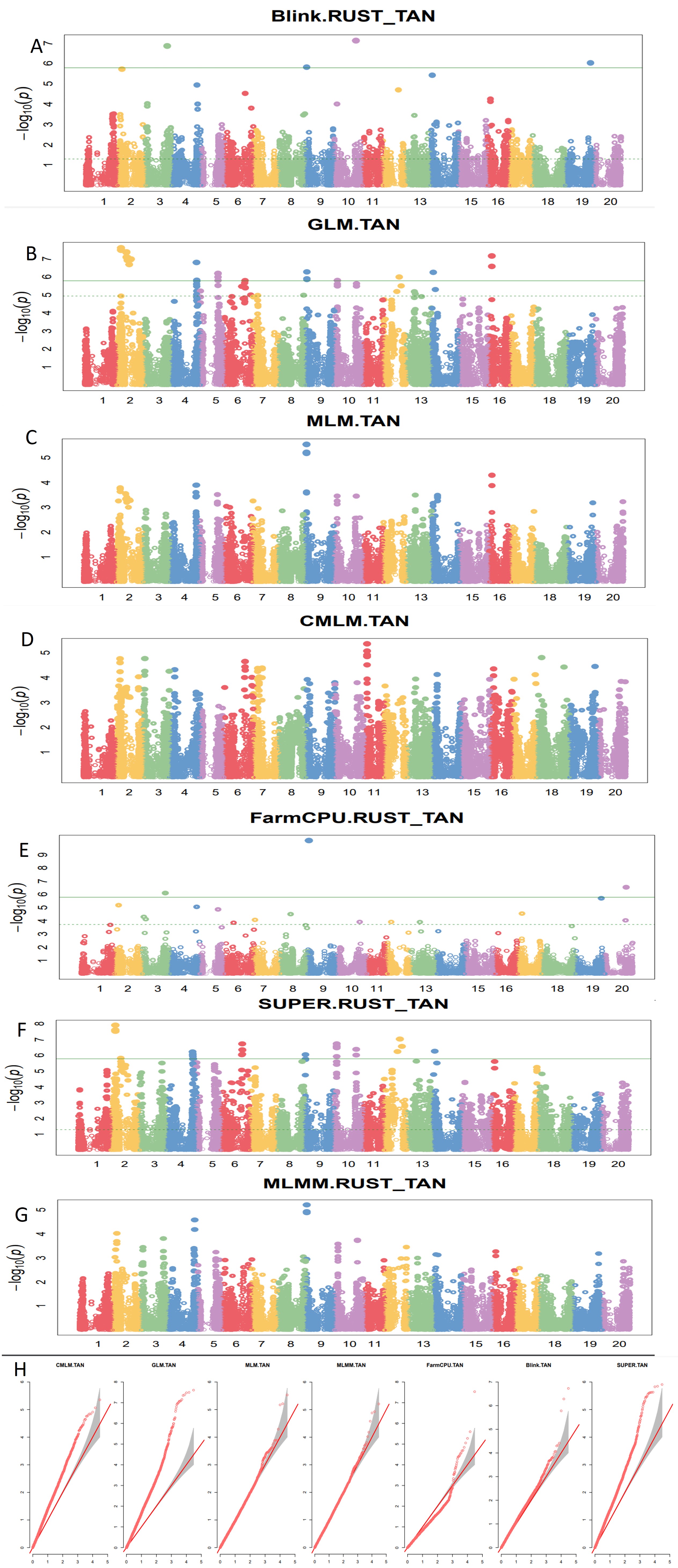

Supplement: Supplementary Figure 2 — The Manhattan plots for SBR by multi-GWAS models: (A) Blink, (B) GLM, (C) MLM, (D) CMLM, (E) FarmCPU, (F) SUPER, (G) MLMM. Additionally: (H) QQ-plots of the above seven models. [file Image_2.jpeg]
