# Supplementary material for: A genome-wide association study and genomic prediction for Phakopsora pachyrhizi resistance in soybean
Source: Front Plant Sci. 2023 May 29;14:1179357. doi: 10.3389/fpls.2023.1179357 (PMC10258334; doi:10.3389/fpls.2023.1179357)
Supplement: Supplementary file 4 [file Table_2.docx]

**S. Table 2: A description of five statistical models for computing genomic estimated breeding value (GEBV)**

| Mothed | Description |
| --- | --- |
| *Ridge regression best linear unbiased predictor (rrBLUP)* | The rrBLUP model is **y = WGβ** + ε, where **y** is the vector phenotype, **β** is the marker effect with β~*N* (0, ***I***σ^2^_β_), **W** is the incidence matrix relating the genotype to the phenotype, **G** is the genetic matrix, and **ε** is the random error.  The solution for the rrBLUP equation is defined by **β** ^= (**Z**^T^**Z** + **I**λ)^-1^**Z**^T^y with **Z** = **WG**. The ridge parameter is described as λ = σ^2^_e_/σ^2^_β_ with σ^2^_e_ being the residual variance and σ^2^_β_ the marker effect variance. rrBLUP is performed using the ‘rrBLUP’ package of R. |
| *Genomic best linear unbiased predictor* *(gBLUP)* | The gBLUP model is **y_r_**= **X**_r_**β** + **Z**_r_µ_r_ + **ε_r_** where the ‘r’ subscript referred to the genotypes involved in the reference panel, **y_r_** is the vector phenotype, β is the genetic effect being assumed to be fixed, **X_r_** is the incidence matrix relating **β** to **y_r_**, µ**_r_** denoted the polygene random additive effect with µ_r_ ~*N*(0, **K**σ^2^_a_), where **K** is the Kinship matrix and σ^2^_a_ the additive genetic variance, ε_r_ is the random error with ε_r_ ~*N*(0, ***I***σ^2^_e_) where ***I*** is an identity matrix and σ^2^_e_ is the residual variance.  The Kinship matrix is divided into reference and inference panel as described by,  $\boldsymbol{K=}\left( \begin{matrix} \boldsymbol{K}_{\boldsymbol{rr}} & & \boldsymbol{K}_{\boldsymbol{ri}} \\ & & \\ \boldsymbol{K}_{\boldsymbol{ir}} & & \boldsymbol{K}_{\boldsymbol{ii}} \end{matrix} \right)$, where **K**_rr_ is the variance-covariance matrix for the reference group, **K**_ii_ represented the variance-covariance matrix for the inference group, and **K**_ir_**=(K**_ri_**)’** denoted the covariance matrix between individuals from the reference and inference groups, respectively.  The predicted genetic effect in the inference panel is obtained using the formula, µ_i_=**K**_ir_(**K**_rr_)^-1^µ_r_, where u_i_ denoted the polygene effect in the inference group, and **K**_ir_, **K**_rr_, and µ_r_ were previously described. gBLUP is performed using GAPIT. |
| *Bayesian least absolute shrinkage and selection operator (Bayesian LASSO)* | Bayesian LASSO is a modified version of LASSO regression. In Bayesian LASSO, posteriors related to the genetic and residual variances were Exponential and Multivariate Normal, respectively. The statistical model is **y** = µ + **Gβ** + **ε** where **y** is the vector phenotype, µ denoted the overall mean, **G** represented the genetic matrix , **β** is the vector of random effect due to SNPs, **ε** represented the vector of random residuals, **β** is defined by **β**\|λ~∏_j_(λ/2)exp{- λ\|**β**_j_\|} with λ~Unif(0,1000000) being the λ prior, and the posterior distribution of ε\|σ^2^_e_~*MVN*(0, ***I****σ^2^_e_*) with σ^2^_e_~Inv-χ^2^(4) being the prior distribution for σ^2^_e_. Bayesian LASSO is done in R using the package ‘BGLR’ with burn-ins and iterations of Markov-Chain Monte Carlo (MCMC) equal to 5,000 and 20,000, respectively. |
| *Random Forest*  *regression* | Random forest regression is based upon on unpruned tree decision. In random forest regression, a new split is obtained from a Bootstrap sample generated from the training set. Splitting at the tree node level is based upon randomly selected subsets of predictors. The prediction of a new observation x((F^_rf_^B^(x)) is the mean outcomes obtained from B trees defined by {T(x, ψ_b_)}_1_^B^. Therefore, the prediction function is described as F^=(1/B)* Σ_b_ T(x, ψ_b_), where b =1….B and ψ_b_ denoted the b^th^ Random Forest tree defined by the split variables, cut-points at each node, and the terminal node. Random Forest regression is established in R using the package ‘Random Forest’. A total of 500 trees and 4 branches were used. |
| *Support Vector Machines* | Support Vector Machines (SVMs) is a kernel-based supervised machine learning approach with a regression equation y = f(X\|**β**) + ε, where f(X\|**β**) = Σ_j_ **β_j_*K_h_* (x, x_j_)** represents the kernel generating function. The inner product kernel ***K*(x_i_, x_j_)** is a N × N symmetric and positive  definite matrix. **β** is vector of weights, typically, only a subset of is nonzero, and the associated observations are called support vectors. In this study, a Gaussian kernel is used. SVMs regression is performed in R using the ‘kernlab’ package. |
